# Behaviour patterns preceding a railway suicide: Explorative study of German Federal Police officers' experiences

**DOI:** 10.1186/1471-2458-11-620

**Published:** 2011-08-04

**Authors:** Karoline Lukaschek, Jens Baumert, Karl-Heinz Ladwig

**Affiliations:** 1 Institute of Epidemiology II, Helmholtz Zentrum München, German Research Centre for Environmental Health, Ingolstädter Landstr. 1, 85764 Neuherberg, Germany; 2 Department of Psychosomatic Medicine and Psychotherapy, Klinikum rechts der Isar, Technische Universität München, Munich, Germany

## Abstract

**Background:**

Constant high-level numbers of railway suicides indicate that prevention strategies against railway suicides are urgently needed. The main question of the present study was whether pre-crash railway suicide behaviour can be identified, using German Federal Police officers experience with suicidal events in railway related environments.

**Methods:**

To collect information on pre-crash railway suicide behaviour, a questionnaire was used and made available on the German Federal Police intranet. A total of 202 subjects (mean age: 41 years, sex: 84.9% male) were included in the analysis. Multivariate logistic regression analyses were performed to predict the prevention of suicide (first model) or demand for counselling (second model) as outcomes. Sex, age, years of service, number of experienced suicides, suicides personally observed, information on suicides obtained from witnesses and finally either counselling/debriefing (first model) or whether officers had prevented a suicide (second model) were used as predictors.

**Results:**

A considerable proportion of police officers reported behavioural patterns preceding a suicide. Half of them observed the dropping or leaving behind of personal belongings or the avoidance of eye contact, more than a third erratic gesture, mimic or movement. Erratic communication patterns and general confusion were each reported by about one quarter. One fifth indicated the influence of alcohol. Less frequently observed behaviour was aimlessly wandering (14.3%) and out of the ordinary clothing (4%). About one third of all railway suicide victims committed suicide in stations. Of those, 70% had chosen an eminent spot. The multivariate logistic regression model using prevented suicides as the outcome identified the number of suicides experienced, counselling/debriefing and having personally observed a suicide as variables with significant impact. The model using counselling/debriefing as the outcome identified age and having prevented a suicide as variables with a significant association.

**Conclusions:**

Our results provide evidence that railway suicides are preceded by identifiable behavioural patterns. This emphasizes the importance of educational efforts, taking into account the knowledge and skills of experienced police officers.

## Background

Suicidal acts carried out in public places, such as railway environments, can be highly traumatic for witnesses [1]. Financial and organisational challenges to the railway system, including delays in service and driver absenteeism, are directly connected with railway suicide incidents [2-4]. Railway suicide prevention remains a challenge across communities in Germany. In an observational period from 1991 to 2000, fatal railway suicides accounted for about 7% of all suicides in Germany, indicating a mean number of fatal incidences of about 17 suicidal events per week on the German Railway net [5]. Higher rates for railway suicide have only been observed in the Netherlands [6,7]. Railway suicide is a particularly violent method of self-destruction, and case fatality is over 90 per cent of all attempts [8]. Nevertheless, prevention of suicidal acts on the railway tracks is possible [2], although not an easy task. Thus, more and well-conceived evidence-based tools to prevent railway suicide are needed.

Gatekeeper training may be one of the most effective approaches to prevent suicide [9]. This training teaches specific groups of people to identify people at high risk for suicide by recognising suicidal risk factors, to assess the levels of risk, and to manage the situation appropriately [10,11]. In a railway environment, gatekeepers must also be familiar with, and alert for, particular vulnerable time windows of excess risk [12]. Until now, to the best of our knowledge, no such gatekeeper concept has been established by any railway company. Officers of the German Federal Police are on duty in railway stations and on tracks of the German railway company "Deutsche Bahn" (DB). Therefore, the likelihood that these officers experienced railway suicides or received first hand reports from witnesses on the scene is high. Thus, they may ideally qualify as gatekeepers. Furthermore, they have experienced suicides, therefore providing a pool of experts, whose knowledge about suicide and behavioural patterns of suicide victims may serve as an educational tool for gatekeeper programmes. The present study sought to collect and evaluate data on police officers' knowledge about and experiences with railway suicide. The main question of the present study was whether pre-crash railway suicide behaviour can be identified.

## Materials and methods

The German Federal Police comprises a total of approximately 41,000 members. Of those, about 33,000 persons are uniformed officers. Among them, 13% are women leading to a male/female ratio of 6.69:1 (all data were obtained by the press office of the German Federal Police on June 23^rd^, 2010). No official figures on the total number of officers working in the police unit formerly known as "Bundesbahnpolizei" (Federal Railway police) were available, but an estimated total of 5,500 officers was communicated to us through personal communication with the press office of the German Federal Police. However, their number may vary greatly due to special time dependent police operations.

Based on peer review and professional commitment, an investigator derived questionnaire (see additional file 1: German Federal Police questionnaire on railway suicides) was developed to assess knowledge and experience of German Federal Police officers with railway suicide in a quantified manner. Previous versions of the questionnaire were tested at the Federal Police Academy Lübeck and critically evaluated by administration staff of the German Federal Ministry of the Interior. The authors took the protection of personal data very seriously and adhered strictly to the regulations of the world medical association declaration of Helsinki and of the German Data Protection Acts (§ 3). For the present study, particulars about personal or factual circumstances of a defined or definable natural person were factually anonymised. The German Federal Ministry of the Interior acted as the ethics committee for the study and questionnaire and approved both. The questionnaire consisted of 27 questions in six modules. The first module contained questions related to the German Federal Police officers' socio-economic status, the number of suicides they had experienced on duty and the counselling or debriefing after such a suicide. In the second module, items were related to the suicide that officers had experienced *most intensely *(source of information, impact of suicide on the officer and whether they had ever prevented suicide). The following modules three and four were related to the suicide victim (age, sex) and the time, date and place (open track, station, eminent spot in station) of the suicide. Finally, in the fifth module, questions were aimed at the observed suicide victims' behavioural patterns such as erratic gesture, mimic or movement, out of the ordinary clothing, aimlessly wandering about, erratic communication pattern, dropping of personal belongings, confused impression, indication of alcohol or the avoidance of eye contact. In a final section, officers were given the possibility to write comments. The questionnaire was made available on the Federal Police Intranet. Officers who had experienced a suicide on duty were asked to fill in the questionnaire. In the questionnaire, we explicitly asked for the suicide which the officer remembered best. Filled questionnaires were returned to the Institute of Epidemiology II, Helmholtz Zentrum München, directly via email or postal mail. Descriptive and multivariate analyses were applied.

A total of 209 subjects returned their filled questionnaires from August to October 2008 and from February to April 2010. The two waves were mainly due to practical and administrative reasons. There was no significant difference between the waves in police officers' sex (p = 0.16), age group (p = 0.27), suicides experienced (p = 0.21) and years of service (p = 0.49). Of the returned questionnaires, seven were excluded from the analysis because the data was unreadable. The remaining 202 subjects (mean age: 41 years, ranging from 19 to 59 years of age) were included in the present analysis (Table 1). However, information on officers' sex was missing in two questionnaires. The majority of the study population was male (n = 169; 84.9%), leading to a male/female ratio of 5.45:1 which was comparable to the overall male/female ratio among police officers of 6.69:1 as described above.

**Table 1:** General description of the German Federal Police Officers' sample (n = 202)

		All
	***Mean***	***95% CI***

Age in years (range: 19-59)	41.04	39.67-42.41
Years of service (range: 0-37)	14.32	12.93-15.71
Suicide events experienced on job (range: 1-300)	17.57	13.43-21.72

	***n***	***%***

Age in years (groups)		
<30	32	15.8
30-39	57	28.2
40-49	62	30.7
>=50	51	25.3
Years of service (groups)		
<=10	85	42.1
11-20	66	32.7
>20	51	25.2
Suicide events experienced on job		
<5	74	36.6
5-20	70	34.7
>20	58	28.7
Counselling was offered	41	20.3
Observed Suicide	14	7
Obtained information by witnesses	150	74.3
Prevented suicide	71	35.15

To analyse the age distribution of the German Federal Police officers, "age" was divided into four groups (in years: < 30, 30-39, 40-49, ≥ 50). To analyse a possible influence of years of service, years of service were divided into three groups (in years: ≤ 10, 11-20, >20). Furthermore, the number of suicides experienced on job was divided into three groups (≤ 5, 6-20, >20), following roughly the tertiles of the distribution.

### Statistical analyses

Bivariate statistical associations were assessed by χ^2 ^test or Fisher's exact test. A multivariate logistic regression analysis with backward elimination variable selection was performed for predicting the prevention of suicide as the outcome. In this model, age, number of suicides experienced on the job, years of service, sex, counselling/debriefing, information about suicide obtained by witnesses and the fact that officers had observed a suicide by themselves were used as predictors.

A multivariate logistic regression analysis with backward elimination variable selection was performed for predicting demands for counselling as the outcome. In this model, age, number of suicides experienced on the job, years of service, sex, whether officers had prevented a suicide, information about suicide obtained by witnesses and the fact that officers had observed a suicide by themselves were used as predictors. For both multivariate logistic regression analyses an entry criterion of a p value < 0.25 in the bivariate analysis and a stay criterion of p value < 0.05 in the end model of the logistic regression was used, following recommendations of Hosmer and Lemeshow [13]. To assess the goodness of fit of the multivariate logistic regression models, we used the c statistic and the Hosmer-Lemeshow Goodness of fit test. For all statistical analyses, a p value less than 0.05 was considered to be statistically significant. All evaluations were performed with the statistical software package R and SAS Version 9.2.

## Results

On average, German Federal Police officers in our study experienced the substantial amount of 17.57 suicides on the job (Table 1). As can be further seen in Table 1, only a minority of all officers (n = 14; 7%) personally observed a suicide by eye contact. The vast majority (n = 150; 74.3%) obtained information about the suicide from witnesses. A relatively large number of officers (n = 71; 35.2%) in the study population reported to have prevented a suicide. German Federal Police officers observed more male (n = 129; 69.4%) than female suicide victims (n = 57; 30.7%), leading to a male/female ratio of 2.26:1. The majority (n = 116; 64.4%) of all reported suicide victims were in the age group 25-60 years. In 16 cases, no information on suicide victim's sex was available.

### Suicide victims' pre-crash behavioural patterns

Approximately 67% of all the reported suicide victims committed suicide on open tracks (Table 2). Of the 33% who committed suicide in stations, however, the vast majority (n = 45; 70%) had chosen an eminent spot in the station (e.g. head or end of station platform; passengers' area). Deviant behaviour may be a relevant indicator of suicidal intent. Indeed, a considerable proportion of officers reported certain behavioural patterns preceding a suicide (see Table 2). Half of them observed the dropping or leaving behind of personal belongings or the avoidance of eye contact. Over a third reported erratic gesture, mimic or movement. Erratic communication patterns and general confusion were observations that were each reported by about one quarter of the respondents. One fifth indicated the influence of alcohol. Less frequently observed behaviour was aimlessly wandering (14.3%) and out of the ordinary clothing (4%).

**Table 2:** Behavioural pattern of suicide victims as observed by the German Federal Police Officers (n = 202)^a^

		Sample		Total
		N	n_total_	%
**Location**	Open track		127	67.2
	Station		64	33.9
	Eminent spot on station		45	25.8
**Deviant**	Erratic gesture, mimic,	184	39	36.8
**behaviour**	movement			
	Out of the ordinary clothing	187	7	4.1
	Aimlessly wandering about	188	13	14.8
	Erratic communication	187	19	26
	pattern			
	Dropping of personal belongings	189	83	50.6
**Impression**	Confused impression	189	19	25
**given**	Indication of Alcohol	188	22	21.4
	Avoidance of eye contact	186	34	50

**^a ^**Sample size N displays the total amount of answers to that question, including "yes", "no" and "unknown". n_total _displays the total number of "yes" answers given by the German Federal Police Officers. Percentages were calculated by using yes/no answers only. To analyse location patterns, we asked for yes *or *no answers. Therefore, results >100% are due to yes *and *no answers.

### Preconditions for successful suicide prevention

A total of 35% (n = 71) of the German Federal Police officers reported to have prevented a suicide during their career. Analysing their reports with respect to officers' and suicide victims' characteristics and behaviour revealed significant associations (χ^2 ^test or Fisher's exact test) compared to the group of officers who had not yet prevented suicide, namely years of service (p = 0.003) and amount of suicides experienced on the job (p = 0.003). Not all variables with a significant univariate association maintained a significant association in the multivariate regression analyses. Multivariate logistic regression analysis with backward elimination strategy identified the number of suicides experienced on the job (p < 0.0001), counselling/debriefing (p = 0.0126) and the fact that officers had observed a suicide by themselves (p = 0.0459) as variables with significant impact on police officers having prevented suicide (Table 3). The model fit was revealed to be sufficient as shown by the c statistic (c = 0.77) and the Hosmer-Lemeshow Goodness of fit test (χ^2 ^= 4.23; p = 0.24).

**Table 3:** Determinants of prevented suicides by multivariate logistic regression analysis with backward elimination variable selection (n = 200)

	OR	95% CI	p value
Number of suicides			<0.0001
experienced < 5	1.00	-	
experienced 5-20	5.08	2.13-12.12	
experienced >20	13.26	5.08-34.60	
Counselling/debriefing	2.85	1.25-6.48	0.013
Suicide observed	4.03	1.03-15.80	0.046

A minority of Police officers (n = 41; 20.3%) stated that they had been counselled or debriefed after a suicide experience. Counselling/debriefing was either performed by a colleague or a pastoral caregiver, or both of them. In one case, a psychiatrist was additionally consulted. Multivariate logistic regression analysis with backward elimination strategy identified age group (p = 0.0071) and having prevented suicide (p = 0.0063) as variables with a significant association on counselling/debriefing (Table 4). The model fit was revealed to be sufficient as shown by the c statistic (p = 0.69) and the Hosmer and Lemeshow Goodness of fit test (χ^2 ^= 3.008; p = 0.699).

**Table 4:** Determinants of counselling/debriefing by multivariate logistic regression analysis with backward elimination variable selection (n = 200)

	OR	95% CI	p value
Age group			0.0071
< 30 years	1.00	-	
30-39 years	4.23	1.49-11.98	
40-49 years	4.89	1.73-13.84	
>= 50 years	5.34	1.76-16.25	
Suicide prevented	2.93	1.36-6.33	0.006

Both grouping strategies resulted in non-significant Hosmer-Lemeshow statistics (p-values 0.24 and 0.699). Thus, the Hosmer-Lemeshow statistic did not indicate disagreement between observed frequencies and predicted probabilities. Moreover, the c statistic values of 0.77 and 0.69 indicated a sufficient capability to discriminate between cases and non-cases. A c statistic above 0.8 is seen as a good model fit. Our models fell slightly short of this value, which may be due to the low response rate to some items.

## Discussion

Information on railway suicide pre-crash behaviour is urgently needed [14]. The first major finding of the present study was that a sample of police officers who were engaged in security operations in the German railway net observed a considerable proportion of suicide events, especially in stations, preceded by specific pre-crash behavioural patterns. These patterns may be valuable clues to identify suicidal intentions. The most prevalent patterns of suicide behaviour observed by more than half of German Police officers who provided information was the dropping or leaving behind of personal belongings (e.g. bags, suicide notes, ID cards) and the avoidance of eye contact. In addition, over a third reported erratic gesture, mimic or movement. Erratic communication patterns (e.g. loudly talking to oneself) and general confusion were each reported by about one quarter. One fifth indicated the influence of alcohol, which was less prevalent than expected [15]. A smaller, yet clinically meaningful proportion of police officers observed aimlessly wandering about (14.8%). Furthermore, a minority of police officers (4%) observed out of the ordinary clothing (e.g. totally dressed in black, extremely bright colours, special items like long, black coats, Stetson or uniforms) which is in contrast to findings in Asia, where Gaylord and Lester [16] frequently observed extraordinary, traditional Chinese clothing style for suicide victims in Hong Kong. Our results are confirmed by smaller studies in this field which achieved comparable results: Among the relevant indicators of suicidal intent reported by Gaylord and Lester [16], who based their analysis on 56 completed suicides and 76 suicide attempts, were sudden droppings of belongings as the train approached, possession of items that ordinarily would be left at home, erratic behaviour, possibly with indication of alcohol/drug intoxication, or conversely over-deliberate moves, the avoidance of eye contact and the manner in which victims dressed.

Additionally, information about suicide intention may also come from the location where suicide was committed in station areas: In our study, from the 64 subjects who committed suicide in the station area, 70% chose an eminent spot (e.g. head of station platform). These results confirm earlier findings by Clarke and Poyner (1994) that about two-thirds of platform incidents occur in the first third of the platform [17].

The reported observations on behavioural patterns preceeding suicide provide important clues on suicidal intention to which security staff must be sensitized in order to prevent suicide. Our findings underscore the value and importance of experience, which apparently leads to alertness and knowledge of approaching procedures in case of being confronted with deviant behavioural patterns. Our results show that German Federal Police officers' preconditions for successful action in suicide prevention and the raising of alertness to deviant behaviour were experience in terms of years of service and numbers of suicides experienced. Furthermore, having personally observed a suicide also had a significant influence. Hence, based on this body of experience, education and training in suicide prevention is possible [2]. Our findings support the necessity of educational efforts in terms of implementing gatekeeper programmes to prevent railway suicides. Recently, Dinkel et al. (2011) analysed three distinctive railway suicide behaviours (jumping, lying, and wandering) which may also be included in gatekeeper programmes [18]. Gatekeeper programmes have been implemented and studied in many populations, including military personnel, clergy, public school staff, peer helpers and clinicians [9,19,20].

In the present study, we could also show an association between counselling/debriefing and police officers' characteristics. Our results show that officers in age groups > 30 years and those who have not prevented suicide did not have counselling. Apparently, older officers are more reluctant to accept counselling services. In reverse, younger officers and those who have prevented suicide are more likely to accept counselling.

Finally, we want to point out that the observed suicide victims male/female ratio of 2.26:1 is comparable to male/female ratio of 2.70:1 as reported by Erazo et al. [12]. The majority (64.4%) of all reported suicide victims were between 25 and 60 years old.

### Limitations of the study

We are aware of the relatively small sample size of our study, compared to the total amount of Federal Police Officers. Furthermore, the former organisation of "Bahnpolizei" (railway police) does not exist anymore. Due to special task forces and shifts in duty, the assignment of personnel to specific organisational structures is not possible. German Federal Police does not keep records about age or sex distributions among the forces, so the numbers we were given were estimates. However, the age and sex distribution of our sample matches the estimated numbers. Unknown factors may bias the answering behaviour of Police officers, with the quality of recollection and recentness as major contributors. The respondents were most likely heterogeneous with regard to the quality of their recollections. Some may have had their experience recently, some perhaps even many years ago. We tried to take this into account by asking for the suicide event which they remember best. Those who have actually experienced suicide on the job are more likely to answer. It might be possible that after a suicide has occurred, witnesses and others try, retrospectively, to look for signs that might have alerted them to the potential for suicide. Besides, the questionnaire was provided on the internet which might be used only by a minority of Police officers. Additionally, respondents can quit in the middle of a web questionnaire easier than in the middle of a face to face interview.

## Conclusion

The results of the present study on German Federal Police officers' experience with railway suicide provide evidence that railway suicides are preceded by identifiable behavioural patterns. This underlines the necessity of educational efforts, taking the knowledge and skills of experienced police officers into account through gatekeeper programmes in order to increase awareness for people with suicide intention and to deepen knowledge of recognisable risk factors. Further research needs to be undertaken to elucidate pre-crash suicidal behaviour and to propose a state of the art intervention, including new tool kits for railway suicide prevention.

## Conflict of interests

The authors declare that they have no competing interests.

## Authors' contributions

KL participated in the design of the study and the acquisition of data, performed the statistical analysis, interpreted the results and drafted the manuscript.

JB helped to perform the statistical analysis and to interpret the results, and revised the manuscript critically.

KHL conceived the study, participated in its design and the interpretation of the results, and revised the manuscript critically.

All authors read and approved the final manuscript.

## Funding

None.

## Pre-publication history

The pre-publication history for this paper can be accessed here:

http://www.biomedcentral.com/1471-2458/11/620/prepub

## Supplementary Material

Additional file 1 **"Questionnaire to collect information on deviant behavior of railway suicide victims", designed by KH Ladwig et al., Helmholtz Zentrum München, German Research Center for Environmental Health**. Click here for file
