# Supplementary material for: Behaviour patterns preceding a railway suicide: Explorative study of German Federal Police officers' experiences
Source: BMC Public Health. 2011 Aug 4;11:620. doi: 10.1186/1471-2458-11-620 (PMC3199597; doi:10.1186/1471-2458-11-620)
Supplement: Additional file 1 — "Questionnaire to collect information on deviant behavior of railway suicide victims", designed by KH Ladwig et al., Helmholtz Zentrum München, German Research Center for Environmental Health. [file 1471-2458-11-620-S1.PDF]

|__|__||__|__| Reg.Nr.

Questionnaire to collect information on deviant behaviour of railway suicide victims

Conducted within the

Suicide Prevention Project of the
German Railway Company
"Deutsche Bahn"

The following questionnaire addresses members of the German federal Police who are on duty in railway stations and on railway tracks and have therefore experienced a railway suicide themselves, or were indirectly exposed to it. Please answer the following questions based on your memory of the event. Naturally, there are no "right" or "wrong" answers. Please answer the questions spontaneously and without pondering them much. All data are completely anonymised before analysis. In case you have experienced more than one suicide, please provide answers regarding the suicide ***you remember most intensely***.

**Personal Details**				
**1**	Age		|__|__| years	
**2**	Sex		☐ 1 male ☐2 female	
**3**	Years of service		|__|__| years	
**4**	To which unit of the Federal police do you belong?		1 ☐ BPOLD BBS 2 ☐ BPOLD B 3 ☐ BPOLD H<br>4 ☐ BPOLD PIR 5 ☐ BPOLD STA 6 ☐ BPOLD KO<br>7 ☐ BPOLD S 8 ☐ BPOLD M 9 ☐ others	
**5**	Suicide events experienced on job		|__|__| Number of suicides	
**6**	Have you ever been offered counsel-ling/follow-up care?	☐1<br>☐2	No, continue with question 8<br>Yes, continue with question 7	
**7**	Who carried out counselling/follow-up care?		1 ☐ colleague 2 ☐ pastoral caregiver 3 ☐ social worker 4 ☐ MD or psychologist 5 ☐ others	
**Questions concerning the railway suicide you *remember best***				
**8**	Did you observe the suicide yourself?	☐1<br>☐2	yes<br>no	

**9**	Did you obtain information about the suicide from witnesses?	☐1<br>☐2	yes<br>no	
**10**	Did the suicidal event emotionally affect you over a long period?		Emotionally affected<br>not at all unbearable<br>☐ - ☐ - ☐ - ☐ - ☐ - ☐ - ☐ - ☐ - ☐ - ☐ - ☐<br>0 1 2 3 4 5 6 7 8 9 10	
**11**	Have you ever been able to prevent a person from committing suicide?	☐1	Yes; Please describe:<br>……………………………………………………………………………<br>……………………………………………………………………………	

**Suicide victim's detail**				
**12**	Sex	☐1<br>☐2<br>☐3	male<br>female<br>unknown	
**13**	Age	☐1<br>☐2<br>☐3<br>☐4	≤ 25 years<br>26 – 60 years<br>> 60 years<br>unknown	
**Time / Place of the suicide**				
**14**	Date		|__|__| |__|__| |__|__||__|__| Day Month Year	
**15**	Time of day	 ☐1<br>☐2<br>☐3<br>☐4<br>☐3<br>☐4	Ca.:<br>6-9 o'clock<br>9-12 o'clock<br>12-15 o'clock<br>15-18 o'clock<br>18-21 o'clock<br>later	

**16**	Open track	☐1<br>☐2	yes<br>no	
**17**	Station area	☐1<br>☐2	yes<br>no	
**18**	Eminent spot on station<br><br>e.g. Head or end of station platform; passengers' area	☐1<br>☐2	yes<br>no	
**18a**	Which eminent spot?		--	
**Deviant behaviour of/impression given by the suicide victim**				
**19**	Erratic gesture, mimic, movement	☐1<br>☐2<br>☐3	Yes<br>No<br>Unknown<br><br>--	
**20**	Out of the ordinary clothing<br><br>Which?	☐1<br>☐2<br>☐3	Yes<br>No<br>Unknown<br><br>---	
**21**	Aimlessly wandering about	☐1<br>☐2<br>☐3	Yes<br>No<br>Unknown	
**22**	Erratic communication pattern (e.g. talking loudly to oneself)	☐1<br>☐2<br>☐3	Yes<br>No<br>Unknown	

**23**	General confused impression	☐1<br>☐2<br>☐3	Yes<br>No<br>Unknown	
**24**	Indication of Alcohol use	☐1<br>☐2<br>☐3	Yes<br>No<br>Unknown	
**25**	Avoidance of eye contact	☐1<br>☐2<br>☐3	Yes<br>No<br>Unknown	
**26**	Dropping of personal belongings	☐1<br>☐2<br>☐3	Yes<br>No<br>Unknown	
**27**	Free text (police officers were given the opportunity to report own experiences in detail or to make suggestions)			

Thank you very much for participating in this important interview! Please check whether you provided an answer to all questions!
